# Supplementary material for: Analysis of PPARγ Signaling Activity in Psoriasis
Source: Int J Mol Sci. 2021 Aug 10;22(16):8603. doi: 10.3390/ijms22168603 (PMC8395241; doi:10.3390/ijms22168603)
Supplement: Supplementary file 1 [file ijms-22-08603-s001.zip › Supplemental materials_Analysis of PPARg signaling activity in psoriasis/Pathway models/Models images and html files/Differentiation of psoriatic T cells/9524.html]

PTGS2


# Protein PTGS2

|  |  |
| --- | --- |
| URN | urn:agi-llid:5743 |
| Total Entities | 0 |
| Connectivity | 15393 |
| Name | PTGS2 |
| Description | prostaglandin-endoperoxide synthase 2 (prostaglandin G/H synthase and cyclooxygenase) |
| Notes | Prostaglandin-endoperoxide synthase (PTGS), also known as cyclooxygenase, is the key enzyme in prostaglandin biosynthesis, and acts both as a dioxygenase and as a peroxidase. There are two isozymes of PTGS: a constitutive PTGS1 and an inducible PTGS2, which differ in their regulation of expression and tissue distribution. This gene encodes the inducible isozyme. It is regulated by specific stimulatory events, suggesting that it is responsible for the prostanoid biosynthesis involved in inflammation and mitogenesis. [provided by RefSeq, Feb 2009] |
| Primary Cell Localization | Plasma membrane |

---

|  |  |
| --- | --- |
| Pathway | Longevity Related Drugs |
|  | Somatostatin Receptors Signaling |
|  | Cortisol in Resolving Inflammation |
|  | Proplatelet Maturation |
|  | Hypothalamic-Pituitary-Adrenal Axis in Acute Phase Response |
|  | Lipoxins and Resolvins in Inflammation Resolution |
|  | Prostaglandin E2 Receptor Signaling in Neurons |
|  | Eicosanoids in Inflammation |
|  | Mast-Cells De Novo Synthesized Mediators via IgE Independent Signaling |
|  | Vascular Endothelial Cell Activation by NO |
|  | Vascular Endothelial Cell Activation by Growth Factors |
|  | Nociception Expression Targets Signaling |
|  | CCR1 Expression Targets |
|  | IL6 Expression Targets -> Nociception |
|  | IL1B Expression Targets -> Nociception |
|  | Prostaglandins Role in Sleep Regulation |
|  | Proteins Involved in Atherosclerosis |
|  | Proteins Involved in Arterial Hypertension |
|  | Arterial Hypertension |
|  | Endothelial Cell Dysfunction in Arterial Hypertension |
|  | Proteins Involved in Pulmonary Hypertension |
|  | Proteins Involved in Myocardial Ischemia |
|  | Preconditioning Ischemia |
|  | Proteins Involved in Myocarditis |
|  | Proteins Involved in Acne Vulgaris |
|  | Hair Follicle Keratinocyte Apoptosis |
|  | Proteins with Altered Expression in Androgenetic Alopecia |
|  | Proteins Involved in Melanoma |
|  | Proteins Involved in Psoriasis |
|  | T-Cells Differentiation Block in Psoriasis |
|  | Proteins Involved in Systemic Lupus Erythematosus |
|  | Genes with Mutations Associated with Vitiligo |
|  | Proteins Involved in Diabetes Mellitus Type 1 |
|  | Proteins Involved in Diabetes Mellitus Type 2 |
|  | Proteins Involved in Diabetic Nephropathy |
|  | Proteins Involved in Diabetic Neuropathy |
|  | Proliferative Diabetic Retinopathy |
|  | Proteins Involved in non-Alcoholic Fatty Liver Disease |
|  | Proteins Involved in Hashimoto's Thyroiditis |
|  | Proteins Involved in Obesity |
|  | Proteins Involved in Colorectal Neoplasms |
|  | Cetuximab Resistance in Colorectal Cancer |
|  | Metastatic Colorectal Cancer |
|  | EGFR Nuclear Signaling in Colorectal Cancer |
|  | Proteins Involved in Inflammatory Bowel Diseases |
|  | Proteins Involved in Helicobacter Infections |
|  | Proteins Involved in Peutz-Jeghers Syndrome |
|  | Proteins Involved in Ulcerative Colitis |
|  | Proteins Involved in Chronic Myeloid Leukemia |
|  | Proteins Involved in Hodgkin Lymphoma |
|  | Proteins Involved in Multiple Myeloma |
|  | Goblet-Cell Related Mucus Secretion in Asthma |
|  | Proteins Involved in Cystic Fibrosis |
|  | Monocytes Function in Gout |
|  | IL1B Induced Arthralgia |
|  | Proteins Involved in Osteoarthritis |
|  | Proteins Involved in Rheumatoid Arthritis |
|  | Proteins Involved in Periodontitis |
|  | Proteins Involved in Osteoporosis |
|  | Microglia Activation in Alzheimer's Disease |
|  | Proteins Involved in Alzheimer's Disease |
|  | Proteins Involved in Epilepsy |
|  | Proteins Involved in Glioblastoma |
|  | Proteins Involved in Glioma |
|  | Dopamine Action in CSD Progression |
|  | Postsynaptic Neuron Activation |
|  | Estradiol Effects in Menstrual Migrane |
|  | Prostaglandin E2 Synthesis Activation via Glutamate in Familial Hemiplegic Migraine |
|  | Microglia Activation in Parkinson's Disease |
|  | Proteins with Altered Expression in Parkinson's Disease |
|  | Proteins Involved in Parkinson's Disease |
|  | Proteins Involved in Amyotrophic Lateral Sclerosis |
|  | Proteins Involved in Multiple Sclerosis |
|  | Proteins Involved in Breast Cancer Related to ERBB2/VEGFR/Akt Signaling Pathway |
|  | Local Estrogen Production in Endometriosis |
|  | Proteins Involved in Endometriosis |
|  | Angiogenesis in Endometriosis |
|  | Proteins with Altered Expression in Endometriosis |
|  | Coagulation Factors and PAR1/2 Receptors (F2R/F2RL1) in Endometriosis |
|  | Proteins Involved in Female Infertility |
|  | Proteins Involved in Spontaneous Abortion |
|  | FOXL2 Mutation in Ovarian Dysgenesis |
|  | BMP Signaling Impairment in Granulosa Cell in POF |
|  | HPV E5 Regulates EDNRA and EGFR Signaling Pathway in Keratinocytes |
|  | Prostate Cancer |
|  | Proteins Involved in Prostate Cancer |
|  | VEGFA in Neovascular Glaucoma |
|  | Proteins Involved in Hearing Loss |
|  | Proteins Involved in Otitis Media |
|  | Proteins Involved in Chronic Obstructive Pulmonary Disease |
|  | Proteins Involved in Cystic Kidney Disease |
|  | Proteins Involved in Glomerulonephritis |
|  | Proteins with Altered Expression in Prostate Cancer |
|  | Sertoli Cells Dysfunction Causes Male Infertility |
|  | HMGB1 Expression Targets |
|  | S100B Expression Targets |
|  | AREG -> NFATC Expression Target |
|  | AREG -> STAT Expression Targets |
|  | AREG -> AP-1 Expression Targets |
|  | Noradrenaline/Gs Expression Targets |
|  | Dopamine/Gs Expression Targets |
|  | Serotonin/Gs Expression Targets |
|  | Noreadrenaline/Gq Expression Targets |
|  | Epinephrine/Gs Expression Targets |
|  | Serotonin/Gq Expression Targets |
|  | Epinephrine/Gq Expression Targets |
|  | Dopamine/Gi Expression Targets |
|  | Aryl Hydrocarbon Receptor Genomic and non-Genomic Signaling |
|  | Aryl Hydrocarbon Receptor/Arachidonic Acid Metabolites Signaling |
|  | BMP2/BMPR2 Expression Targets |
|  | Receptor Tyrosine Kinase Family -> Expression Targets in Bone |
|  | GPCRs Family -> Expression Targets in Bone |
|  | Elevated Receptors -> Expression Targets in Bone and Joint |
|  | Elevated Receptors -> Expression Targets in Bone |
|  | CD80 -> STAT Expression Targets |
|  | CD81 Expression Targets |
|  | CD80 -> NF-kB Expression Targets |
|  | CD80 -> NFATC Expression Targets |
|  | CD80 -> ATF/CREB/CREBBP Expression Targets |
|  | CTF1 Expression Targets |
|  | Elevated Receptors -> Expression Targets in Blood vessel |
|  | Elevated Receptors -> Expression Targets in Blood Vessel |
|  | CXCL1 Expression Targets |
|  | CXCL5 Expression Targets |
|  | CXCL12 Expression Targets |
|  | CCL3 Expression Targets |
|  | CCL7 Expression Target |
|  | IL8 Expression Targets |
|  | CSF3 Expression Targets |
|  | CSF2 -> NF-kB Expression Targets |
|  | CSF2 -> STAT Expression Targets |
|  | LIF Expression Targets |
|  | CNTF Expression Targets |
|  | Valdecoxib Induced Ischemic Disease |
|  | Clozapine Induced Granulocytopenia |
|  | Cyclosporine Induced Nephrotoxicity |
|  | EGF -> CTNN Expression Targets |
|  | EGF -> HIF1A Expression Targets |
|  | EGF -> AP-1/ATF Expression Targets |
|  | EGF -> CREB/CREBBP/ELK/SRF/MYC Expression Targets |
|  | EGF -> MEF/MYOD/NFATC Expression Targets |
|  | EGF -> TP53 Expression Targets |
|  | EGF -> STAT Expression Targets |
|  | EREG -> STAT Expression Targets |
|  | EREG -> AP-1/ATF Expression Targets |
|  | Elevated Receptors -> Expression Targets in Endocrine Gands |
|  | F2 -> AP-1/CREB/ELK/SRF/SP1 Expression Targets |
|  | F2 -> STAT1/NF-kB Expression Targets |
|  | PLG -> AP-1/CREB/ELK/SRF/SP1 Expression Targets |
|  | PLG -> STAT1/NF-kB Expression Targets |
|  | FGF10 -> AP-1/CREB/CREBBP/MYC Expression Targets |
|  | FGF2 -> STAT Expression Targets |
|  | FGF10 -> STAT Expression Targets |
|  | FGF7 -> AP-1/CREB/CREBBP/MYC Expression Targets |
|  | FGF2 -> AP-1/CREB/CREBBP/ELK/SRF/MYC Expression Targets |
|  | ADCYAP1 Expression Targets |
|  | FSHR Expression Targets |
|  | Glutamate/Gq Expression Targets |
|  | Elevated Receptors -> Expression Targets in Mouth |
|  | Elevated Receptors -> Expression Targets in Colon |
|  | GH1/GHR -> STAT Expression Targets |
|  | HBEGF -> STAT Expression Targets |
|  | HBEGF -> AP-1/ATF Expression Targets |
|  | HGF -> STAT Expression Targets |
|  | HGF -> AP-1/CREB/ELK/SRF/MYC Expression Targets |
|  | STAT3 and NFkB Activate Inflammation-Induced Tumorigenesis |
|  | IL23A and IL17A Provoke Cancer-Associated Inflammation |
|  | HIF1A in Vasculogenic Mimicry of Cancer |
|  | Integrins in Cancer Cell Motility, Invasion and Survival |
|  | IGF1 -> MEF/MYOD/MYOG Expression Targets |
|  | IGF2 -> STAT Expression Targets |
|  | IGF1 -> STAT Expression Targets |
|  | IGF2 -> HIF1A/MYC Expression Targets |
|  | IGF1 -> ELK/SRF/HIF1A/MYC/SREBF Expression Targets |
|  | Fibronectin Expression Targets |
|  | ICAM1 Expression Targets |
|  | EGFR -> Expression Targets in Skin |
|  | IFNG/IFNR Expression Targets |
|  | IL13 Expression Targets |
|  | IL6 Expression Targets |
|  | IL4 Expression Targets |
|  | IL2 Expression Targets |
|  | Leptin -> STAT Expression Targets |
|  | Leptin -> ELK/SRF Expression Targets |
|  | Prostaglandin F Expression Targets |
|  | Thromboxane A2 Expression Targets |
|  | LPA Expression Targets |
|  | PAF/Gq -> AP-1/ATF1/CREB/ERK/SRF Expression Targets |
|  | Dronabinol/Anandamide Expression Targets |
|  | Morphine Expression Targets |
|  | PAF/Gq -> NF-kB Expression Targets |
|  | S1P Expression Targets |
|  | PGE1 Expression Targets |
|  | Omega-6-Fatty Acid Metabolism |
|  | Proteins/Ions Transporters -> Expression Targets in Lymphoid System and Blood |
|  | TGF-beta Receptors Family -> Expression Targets in Lymphoid System and Blood |
|  | EGFR-> Expression Targets in Lymphoid System and Blood |
|  | Elevated Receptors -> Expression Targets in Lymphoid System and Blood |
|  | CD Markers -> Expression Targets in Lymphoid System and Blood |
|  | GPCRs Family -> Expression Targets in Lymphoid System and Blood |
|  | Immunoglobulin Superfamily -> Expression Targets in Lymphoid System and Blood |
|  | NRG1 -> EP300/ETS/ETV/SP1 Expression Targets |
|  | NRG1 -> CREB/CREBBP/ELK/SRF/MYC Expression Targets |
|  | NRG1/Catenin Expression Targets |
|  | NRG1 -> STAT Expression Targets |
|  | NRG1 -> AP-1/ATF Expression Targets |
|  | PTGER4 -> Expression Targets in Nerve Tissue |
|  | P2RX7 -> Expression Targets in Nerve Tissue |
|  | Elevated Receptors -> Expression Targets in Nerve Tissue |
|  | NGF -> SMAD3/NF-kB Expression Targets |
|  | NGF -> AP-1/TP53/MYC Expression Targets |
|  | NGF -> FOXO/MYCN/ELK-SRF Expression Targets |
|  | NGF -> CREB/CEBPB/MEF2A Expression Targets |
|  | DLL1 Expression Targets |
|  | NOTCH Expression Targets |
|  | Adenosine Expression Targets |
|  | OSM/OSMR Expression Targets |
|  | PDGF -> AP-1/CREB/CREBBP/MYC Expression Targets |
|  | PDGF -> STAT Expression Targets |
|  | AVP/Gs -> CREB/ELK/SRF/AP-1/EGR Expression Targets |
|  | GNRH1 Expression Targets |
|  | NTS Expression Targets |
|  | GAST Expression Targets in GAST Expression Targets |
|  | AGT -> TP53 Expression Targets |
|  | GNRH2 Expression Targets |
|  | OXT Expression Targets |
|  | EDN1 Expression Targets |
|  | AGT -> CREB Expression Targets |
|  | SST Expression Targets |
|  | AVP/Gs -> MEF/MYOD/NFATC/MYOG Expression Targets |
|  | AVP/Gq -> STAT Expression Targets |
|  | TAC1 Expression Targets |
|  | AVP/Gs -> STAT Expression Targets |
|  | AVP/Gq -> MEF/MYOD/NFATC/MYOG Expression Targets |
|  | EDN3 Expression Targets |
|  | AGT -> STAT Expression Targets |
|  | AVP/Gq -> CREB/ELK/SRF/AP-1/EGR Expression Targets |
|  | PRL/PRLR Expression Targets |
|  | PRL/GHR -> STAT Expression Targets |
|  | PRL/GHR -> NF/kB/ELK/SRF/MYC Expression Targets |
|  | KITLG -> STAT Expression Targets |
|  | KITLG -> AP-1/CREB/CREBBP/MYC Expression Targets |
|  | PAF Expression Targets |
|  | Elevated Receptors -> Expression Targets in Seminal Vesicle |
|  | AMPK Signaling |
|  | VEGF Signaling |
|  | Androgen Receptor Genomic Signaling |
|  | TCR -> NFAT Expression Targets |
|  | TCR -> STAT Expression Targets |
|  | TCR -> AP-1 Expression Targets |
|  | TGFA -> STAT Expression Targets |
|  | TGFA -> CTNNB/CTNND Expression Targets |
|  | TGFA -> TP53 Expression Targets |
|  | TGFA -> HIF1A Expression Targets |
|  | TGFA -> MEF/MYOD/NFATC Expression Targets |
|  | TGFA/AP-1/ATF Expression Targets |
|  | TGFA -> CREB/CREBBP/ELK-SRF/MYC Expression Targets |
|  | TGFB2-TGFBR2 Expression Targets |
|  | TGFB1-TGFBR2 Expression Targets |
|  | TGFB1-TGFBR1/AP-1 Expression Targets |
|  | TGFB1-ACVRL1 Expression Targets |
|  | FASLG Expression Targets |
|  | TNF -> NF-kB Expression Targets |
|  | TNF -> AP-1 Expression Targets |
|  | CD72 -> AP-1 Expression Targets |
|  | CD40LG -> STAT Expression Targets |
|  | CD40LG -> NF-kB/ELK/SRF -> CREB/NFATC Expression Targets |
|  | TNF -> CREB Expression Targets |
|  | TNF -> STAT Expression Targets |
|  | TNF -> TP53/ATF Expression Targets |
|  | TLR3 Expression Targets |
|  | TLR4 -> AP-1 Expression Targets |
|  | TLR5 Expression Targets |
|  | TLR7 Expression Targets |
|  | IL1B -> NO Expression Targets |
|  | IL1B -> PGE2 Expression Targets |
|  | TLR1 -> 2/6 Expression Targets |
|  | IL1B Expression Targets |
|  | TLR4 -> NF-kB/IRF Expression Targets |
|  | IL1A Expression Targets |
|  | TLR9 Expression Targets |
|  | ROS in Triggering Vascular Inflammation |
|  | ROS and RNS in Vasoconstriction and Vasodilation Regulation |
|  | Elevated Receptors -> Expression Targets in Kidney |
|  | VEGFA -> ATF/CREB/ELK-SRF Expression Targets |
|  | VEGFA -> NFATC Expression Targets |
|  | VEGFA -> STAT Expression Targets |
|  | VEGFA -> AP-1/CREBBP/MYC Expression Targets |
|  | VEGFA -> CTNNB/CTNND Expression Targets |
|  | WNT1 Expression Targets |
|  | WNT5A Expression Targets |
|  | PPAR Psoriasis |
|  | prarg neg,ukn expres targets, ps-positive |
|  | PPARG negative targets |
|  | pparg expr target\_regulators |
|  | Neighbors of disease exacerbation |
|  | Neighbors of tumor progression |
|  | Neighbors of cancer progression |
|  | Neighbors of inflammatory status |
|  | Neighbors of overall survival |
|  | Neighbors of disease severity |
|  | Neighbors of tumor size |
|  | Neighbors of lesion size |
|  | Neighbors of mortality |
|  | Neighbors of survival rate |
|  | Neighbors of infarct size |
|  | Neighbors of survival time |
|  | Neighbors of functional recovery |
|  | Neighbors of insulin sensitivity |
|  | Neighbors of bacterial load |
|  | Neighbors of cancer survival |
|  | Neighbors of graft survival |
|  | Neighbors of therapeutic efficacy |
|  | Neighbors of clinical stage |
|  | Neighbors of lethality |
|  | Neighbors of tumor regression |
|  | Neighbors of recurrence-free survival |
|  | Neighbors of disease-free survival |
|  | Neighbors of tumor response |
|  | Neighbors of long-term survival |
|  | Neighbors of treatment outcome |
|  | Neighbors of bone mass |
|  | Neighbors of body weight |
|  | Neighbors of body weight gain |
|  | Neighbors of cardiovascular risk |
|  | Neighbors of microvessel density |
|  | Neighbors of tumor recurrence |
|  | Neighbors of tumor promotion |
|  | Neighbors of neutrophil count |
|  | Neighbors of glucose tolerance |
|  | Neighbors of morbidity |
|  | Neighbors of fibrosis progression |
|  | Neighbors of mortality rate |
|  | Neighbors of exhaustion |
|  | Neighbors of bone mineral density |
|  | Neighbors of adverse outcome |
|  | Neighbors of radioresistance |
|  | Neighbors of lesion area |
|  | Neighbors of cell density |
|  | Neighbors of cancer-specific survival |
|  | Neighbors of progression-free survival |
|  | Neighbors of apoptosis rate |
|  | Neighbors of early mortality |
|  | Neighbors of shear stress |
|  | Neighbors of viral load |
|  | Neighbors of blood pressure |
|  | Neighbors of leukocyte count |
|  | Neighbors of tumor grade |
|  | Neighbors of vascular reactivity |
|  | Neighbors of vascular leakage |
|  | Neighbors of body mass index |
|  | Neighbors of vascular density |
|  | Neighbors of muscle mass |
|  | Neighbors of cancer risk |
|  | Neighbors of cancer incidence |
|  | Neighbors of tumor aggressiveness |
|  | Neighbors of systolic blood pressure |
|  | Neighbors of tumor resistance |
|  | Neighbors of blood glucose |
|  | Neighbors of ejection fraction |
|  | Neighbors of median survival |
|  | Neighbors of renal hypertrophy |
|  | Neighbors of fasting plasma glucose |
|  | Neighbors of osteoclast number |
|  | Neighbors of cancer recurrence |
|  | Neighbors of antimetastatic activity |
|  | Neighbors of energy expenditure |
|  | Neighbors of vascular tone |
|  | Neighbors of radiosensitivity |
|  | Neighbors of breast cancer risk |
|  | Neighbors of proliferation index |
|  | Neighbors of functional status |
|  | Neighbors of gastric cancer risk |
|  | Neighbors of neurological outcome |
|  | Neighbors of fertility |
|  | Neighbors of recurrence rate |
|  | Neighbors of pathological response |
|  | Neighbors of skin thickness |
|  | Neighbors of insulin level |
|  | Neighbors of bone volume |
|  | Neighbors of fibroblast phenotype |
|  | Neighbors of disease exacerbation |
|  | Neighbors of tumor progression |
|  | Neighbors of cancer progression |
|  | Neighbors of inflammatory status |
|  | Neighbors of overall survival |
|  | Neighbors of disease severity |
|  | Neighbors of tumor size |
|  | Neighbors of lesion size |
|  | Neighbors of mortality |
|  | Neighbors of survival rate |
|  | Neighbors of infarct size |
|  | Neighbors of survival time |
|  | Neighbors of functional recovery |
|  | Neighbors of insulin sensitivity |
|  | Neighbors of bacterial load |
|  | Neighbors of cancer survival |
|  | Neighbors of graft survival |
|  | Neighbors of therapeutic efficacy |
|  | Neighbors of clinical stage |
|  | Neighbors of lethality |
|  | Neighbors of tumor regression |
|  | Neighbors of recurrence-free survival |
|  | Neighbors of disease-free survival |
|  | Neighbors of tumor response |
|  | Neighbors of long-term survival |
|  | Neighbors of treatment outcome |
|  | Neighbors of bone mass |
|  | Neighbors of body weight |
|  | Neighbors of body weight gain |
|  | Neighbors of cardiovascular risk |
|  | Neighbors of microvessel density |
|  | Neighbors of tumor recurrence |
|  | Neighbors of tumor promotion |
|  | Neighbors of neutrophil count |
|  | Neighbors of glucose tolerance |
|  | Neighbors of morbidity |
|  | Neighbors of fibrosis progression |
|  | Neighbors of mortality rate |
|  | Neighbors of exhaustion |
|  | Neighbors of bone mineral density |
|  | Neighbors of adverse outcome |
|  | Neighbors of radioresistance |
|  | Neighbors of lesion area |
|  | Neighbors of cell density |
|  | Neighbors of cancer-specific survival |
|  | Neighbors of progression-free survival |
|  | Neighbors of apoptosis rate |
|  | Neighbors of early mortality |
|  | Neighbors of shear stress |
|  | Neighbors of viral load |
|  | Neighbors of blood pressure |
|  | Neighbors of leukocyte count |
|  | Neighbors of tumor grade |
|  | Neighbors of vascular reactivity |
|  | Neighbors of vascular leakage |
|  | Neighbors of body mass index |
|  | Neighbors of vascular density |
|  | Neighbors of muscle mass |
|  | Neighbors of cancer risk |
|  | Neighbors of cancer incidence |
|  | Neighbors of tumor aggressiveness |
|  | Neighbors of systolic blood pressure |
|  | Neighbors of tumor resistance |
|  | Neighbors of blood glucose |
|  | Neighbors of ejection fraction |
|  | Neighbors of median survival |
|  | Neighbors of renal hypertrophy |
|  | Neighbors of fasting plasma glucose |
|  | Neighbors of osteoclast number |
|  | Neighbors of cancer recurrence |
|  | Neighbors of antimetastatic activity |
|  | Neighbors of energy expenditure |
|  | Neighbors of vascular tone |
|  | Neighbors of radiosensitivity |
|  | Neighbors of breast cancer risk |
|  | Neighbors of proliferation index |
|  | Neighbors of functional status |
|  | Neighbors of gastric cancer risk |
|  | Neighbors of neurological outcome |
|  | Neighbors of fertility |
|  | Neighbors of recurrence rate |
|  | Neighbors of pathological response |
|  | Neighbors of skin thickness |
|  | Neighbors of insulin level |
|  | Neighbors of bone volume |
|  | Neighbors of fibroblast phenotype |
|  | Neighbors of cell development |
|  | Neighbors of immune response |
|  | Neighbors of cell population |
|  | Neighbors of T-cell development |
|  | Neighbors of inflammatory response |
|  | Neighbors of wound healing |
|  | Neighbors of angiogenesis |
|  | Neighbors of innate immune response |
|  | Neighbors of cell function |
|  | Neighbors of macrophage activation |
|  | Neighbors of cell formation |
|  | Neighbors of adaptive immune response |
|  | Neighbors of osteoclast development |
|  | Neighbors of epithelial to mesenchymal transition |
|  | Neighbors of immunity |
|  | Neighbors of cell proliferative response |
|  | Neighbors of chemotaxis |
|  | Neighbors of dendritic cell differentiation |
|  | Neighbors of cell survival |
|  | Neighbors of osteoclast differentiation |
|  | Neighbors of cell phenotype |
|  | Neighbors of T-cell proliferation |
|  | Neighbors of tissue remodeling |
|  | Neighbors of cell adhesion |
|  | Neighbors of T-cell activation |
|  | Neighbors of endothelial cell proliferation |
|  | Neighbors of fibrogenesis |
|  | Neighbors of tumor growth |
|  | Neighbors of dendritic cell development |
|  | Neighbors of tissue repair |
|  | Neighbors of cell count |
|  | Neighbors of cell infiltration |
|  | Neighbors of cell interaction |
|  | Neighbors of SMC proliferation |
|  | Neighbors of lumen formation |
|  | Neighbors of endothelial cell migration |
|  | Neighbors of adipogenesis |
|  | Neighbors of lipid storage |
|  | Neighbors of cell damage |
|  | Neighbors of pregnancy |
|  | Neighbors of monocyte differentiation |
|  | Neighbors of aging |
|  | Neighbors of cell motility |
|  | Neighbors of bone resorption |
|  | Neighbors of monocyte migration |
|  | Neighbors of fibroblast proliferation |
|  | Neighbors of adipocyte differentiation |
|  | Neighbors of macrophage migration |
|  | Neighbors of microglial activation |
|  | Neighbors of neutrophil recruitment |
|  | Neighbors of regeneration |
|  | Neighbors of cell migration |
|  | Neighbors of cancer growth |
|  | Neighbors of stem cell proliferation |
|  | Neighbors of smooth muscle cell migration |
|  | Neighbors of hepatic regeneration |
|  | Neighbors of transendothelial migration |
|  | Neighbors of phagocytosis |
|  | Neighbors of immune system activation |
|  | Neighbors of leukocyte migration |
|  | Neighbors of leukocyte recruitment |
|  | Neighbors of osteoclast formation |
|  | Neighbors of monocyte recruitment |
|  | Neighbors of cell invasion |
|  | Neighbors of heart function |
|  | Neighbors of hemopoiesis |
|  | Neighbors of bone remodeling |
|  | Neighbors of ossification |
|  | Neighbors of T-cell function |
|  | Neighbors of immune system function |
|  | Neighbors of lung development |
|  | Neighbors of autophagy |
|  | Neighbors of monocyte adhesion |
|  | Neighbors of transcription activation |
|  | Neighbors of senescence |
|  | Neighbors of T-cell response |
|  | Neighbors of tumor immunity |
|  | Neighbors of hepatocyte proliferation |
|  | Neighbors of neuroprotection |
|  | Neighbors of stem cell differentiation |
|  | Neighbors of endothelial cell function |
|  | Neighbors of neuronal death |
|  | Neighbors of cell growth |
|  | Neighbors of ROS generation |
|  | Neighbors of epithelial cell proliferation |
|  | Neighbors of first trimester pregnancy |
|  | Neighbors of cancer cell growth |
|  | Neighbors of sensitization |
|  | Neighbors of hemato-encephalic barrier |
|  | Neighbors of macrophage function |
|  | Neighbors of cellular immune response |
|  | Neighbors of cellular senescence |
|  | Neighbors of colony formation |
|  | Neighbors of macrophage differentiation |
|  | Neighbors of cell differentiation |
|  | Neighbors of keratinocyte proliferation |
|  | Neighbors of life span |
|  | Neighbors of cell homeostasis |
|  | Neighbors of psoriasis |
|  | Neighbors of inflammatory disease |
|  | Neighbors of rheumatoid arthritis |
|  | Neighbors of atherosclerosis |
|  | Neighbors of fibrosis |
|  | Neighbors of colitis |
|  | Neighbors of arthritis |
|  | Neighbors of inflammatory bowel disease |
|  | Neighbors of injury |
|  | Neighbors of atherogenesis |
|  | Neighbors of vascular remodeling |
|  | Neighbors of metastasis |
|  | Neighbors of carcinogenesis |
|  | Neighbors of multiple sclerosis |
|  | Neighbors of chronic inflammation |
|  | Neighbors of inflammation |
|  | Neighbors of tumor microenvironment |
|  | Neighbors of pathological angiogenesis |
|  | Neighbors of obesity |
|  | Neighbors of vascular disease |
|  | Neighbors of diabetes mellitus |
|  | Neighbors of asthma |
|  | Neighbors of pneumonia |
|  | Neighbors of hyperplasia |
|  | Neighbors of osteoarthritis |
|  | Neighbors of liver fibrosis |
|  | Neighbors of chronic obstructive pulmonary disease |
|  | Neighbors of myocardial infarction |
|  | Neighbors of leukocyte infiltration |
|  | Neighbors of reperfusion injury |
|  | Neighbors of airway inflammation |
|  | Neighbors of hepatitis |
|  | Neighbors of insulin resistance |
|  | Neighbors of infection |
|  | Neighbors of vascular injury |
|  | Neighbors of renal injury |
|  | Neighbors of preeclampsia |
|  | Neighbors of macrophage infiltration |
|  | Neighbors of liver injury |
|  | Neighbors of neuroinflammation |
|  | Neighbors of sepsis |
|  | Neighbors of death |
|  | Neighbors of type 1 diabetes |
|  | Neighbors of encephalomyelitis |
|  | Neighbors of autoimmune disease |
|  | Neighbors of ischemic stroke |
|  | Neighbors of dermatitis |
|  | Neighbors of neutrophil infiltration |
|  | Neighbors of heart disease |
|  | Neighbors of diabetic nephropathy |
|  | Neighbors of lung injury |
|  | Neighbors of immunopathology |
|  | Neighbors of hepatocellular carcinoma |
|  | Neighbors of autoimmunity |
|  | Neighbors of acute lung injury |
|  | Neighbors of gastric cancer |
|  | Neighbors of atherosclerotic plaque |
|  | Neighbors of experimental autoimmune encephalomyelitis |
|  | Neighbors of colorectal cancer |
|  | Neighbors of cardiac remodeling |
|  | Neighbors of breast cancer |
|  | Neighbors of ischemia |
|  | Neighbors of systemic lupus erythematosus |
|  | Neighbors of melanoma |
|  | Neighbors of collagen-induced arthritis |
|  | Neighbors of cartilage degeneration |
|  | Neighbors of interstitial fibrosis |
|  | Neighbors of neoplasm invasion |
|  | Neighbors of kidney disease |
|  | Neighbors of heart failure |
|  | Neighbors of lung disease |
|  | Neighbors of pulmonary fibrosis |
|  | Neighbors of cardiovascular disease |
|  | Neighbors of stroke |
|  | Neighbors of cancer |
|  | Neighbors of cerebral ischemia |
|  | Neighbors of type 2 diabetes |
|  | Neighbors of neoplasm |
|  | Neighbors of endometriosis |
|  | Neighbors of neointima |
|  | Neighbors of vasculitis |
|  | Neighbors of atrophy |
|  | Neighbors of diet-induced obesity |
|  | Neighbors of respiratory hypersensitivity |
|  | Neighbors of myocardial fibrosis |
|  | Neighbors of synovitis |
|  | Neighbors of coronary artery disease |
|  | Neighbors of rupture |
|  | Neighbors of endothelial cell dysfunction |
|  | Neighbors of liver disease |
|  | Neighbors of prostate cancer |
|  | Neighbors of hypertrophy |
|  | Neighbors of osteolysis |
|  | Neighbors of ovarian cancer |
|  | Neighbors of pancreatic cancer |
|  | Neighbors of hypertension |
|  | Neighbors of intimal hyperplasia |
|  | Neighbors of allograft rejection |
|  | Neighbors of proteinuria |
|  | Neighbors of epithelial cell |
|  | Neighbors of keratinocyte |
|  | Neighbors of vascular smooth muscle cell |
|  | Neighbors of umbilical vein endothelial cell |
|  | Neighbors of monocyte |
|  | Neighbors of alveolar macrophage |
|  | Neighbors of synoviocyte |
|  | Neighbors of macrophage |
|  | Neighbors of adipocyte |
|  | Neighbors of fibroblast |
|  | Neighbors of airway epithelial cell |
|  | Neighbors of PBMC |
|  | Neighbors of stromal cell |
|  | Neighbors of osteoblast |
|  | Neighbors of endometrium cell |
|  | Neighbors of microglia |
|  | Neighbors of trophoblast |
|  | Neighbors of intestine epithelium cell |
|  | Neighbors of mononuclear cell |
|  | Neighbors of hepatic stellate cell |
|  | Neighbors of kidney mesangial cell |
|  | Neighbors of endothelial cell |
|  | Neighbors of dendritic cell |
|  | Neighbors of Kuppfer cell |
|  | Neighbors of inflammatory cell |
|  | Neighbors of bone marrow macrophage |
|  | Neighbors of peritoneal macrophage |
|  | Neighbors of neutrophil |
|  | Neighbors of smooth muscle myocyte |
|  | Neighbors of astrocyte |
|  | Neighbors of lung fibroblast |
|  | Neighbors of mononuclear phagocyte |
|  | Neighbors of capillary endothelial cell |
|  | Neighbors of immunocompetent cell |
|  | Neighbors of granulocyte |
|  | Neighbors of cardiac myocyte |
|  | Neighbors of cancer-associated fibroblast |
|  | Neighbors of myeloid cell |
|  | Neighbors of mesenchymal stroma cell |
|  | Neighbors of stem cell |
|  | Neighbors of tumor infiltrating macrophage |
|  | Neighbors of B-cell |
|  | Neighbors of granulosa cell |
|  | Neighbors of leukocyte |
|  | Neighbors of chondrocyte |
|  | Neighbors of mesenchymal cell |
|  | Neighbors of T-cell |
|  | Neighbors of myofibroblast |
|  | Neighbors of pancreatic stellate cell |
|  | Neighbors of mast cell |
|  | Neighbors of spleen cell |
|  | Neighbors of neuroglia |
|  | Neighbors of antigen-presenting cell |
|  | Neighbors of epidermis cell |
|  | Neighbors of bone marrow derived mast cell |
|  | Neighbors of brain cell |
|  | Neighbors of retina cell |
|  | Neighbors of peripheral lymphocyte |
|  | Neighbors of lung |
|  | Neighbors of liver |
|  | Neighbors of kidney |
|  | Neighbors of skin |
|  | Neighbors of intestine |
|  | Neighbors of lymph node |
|  | Neighbors of colon |
|  | Neighbors of airway |
|  | Neighbors of blood vessel |
|  | Neighbors of heart |
|  | Neighbors of aorta |
|  | Neighbors of gastrointestinal tract |
|  | Neighbors of microvessel |
|  | Neighbors of bone |
|  | Neighbors of placenta |
|  | Neighbors of carotid artery |
|  | Neighbors of uterus |
|  | Neighbors of esophagus |
|  | Neighbors of periodontium |
|  | Neighbors of brain |
|  | Neighbors of ovary |
|  | Neighbors of breast |
|  | Neighbors of blood vessel wall |
|  | Neighbors of stomach |
|  | Neighbors of artery |
|  | Neighbors of cardiovascular system |
|  | Neighbors of pancreas |
|  | Neighbors of prostate |
|  | Neighbors of central nervous system |
|  | Neighbors of kidney glomerulus |
|  | Neighbors of lymphatic system |
|  | Neighbors of colorectal region |
|  | Neighbors of alveolus |
|  | Neighbors of uterine cervix |
|  | Neighbors of arteriole |
|  | Neighbors of villus |
|  | Neighbors of nose |
|  | Neighbors of eye |
|  | Neighbors of hippocampus |
|  | Neighbors of intervertebral disc |
|  | Neighbors of mammary gland |
|  | Neighbors of cerebral cortex |
|  | Neighbors of brain blood vessel |
|  | Neighbors of bladder |
|  | Neighbors of nervous system |
|  | Neighbors of blastocyst |
|  | Neighbors of retina blood vessel |
|  | Neighbors of mesentery |
|  | Neighbors of corpus luteum of ovary |
|  | Neighbors of small intestine |
|  | Neighbors of knee joint |
|  | Neighbors of lymphatic vessels |
|  | Neighbors of choroid plexus |
|  | Neighbors of NF-kB family |
|  | Neighbors of oxidized LDL |
|  | Neighbors of JNK |
|  | Neighbors of IL1 family |
|  | Neighbors of PPAR |
|  | Neighbors of Jun/Fos |
|  | Neighbors of NFKBI |
|  | Neighbors of PKC |
|  | Neighbors of PI3K |
|  | Neighbors of STAT family |
|  | Neighbors of mitogen-activated protein kinase |
|  | Neighbors of IFNAR ligand |
|  | Neighbors of transforming growth factor |
|  | Neighbors of TLR |
|  | Neighbors of cytokine |
|  | Neighbors of interferon |
|  | Neighbors of interleukin |
|  | Neighbors of JAK |
|  | Neighbors of MEK1/2 |
|  | Neighbors of PDGF |
|  | Neighbors of Ras GTPase |
|  | Neighbors of ERK1/2 |
|  | Neighbors of growth factor |
|  | Neighbors of PKA |
|  | Neighbors of ovalbumin |
|  | Neighbors of LDL |
|  | Neighbors of NF-AT family |
|  | Neighbors of beta adrenoceptor |
|  | Neighbors of chorionic gonadotropin |
|  | Neighbors of IKBK |
|  | Neighbors of adipokine |
|  | Neighbors of protein tyrosine kinase |
|  | Neighbors of NAD(P)H oxidase |
|  | Neighbors of IgG |
|  | Neighbors of SMAD subfamily |
|  | Neighbors of AMPK |
|  | Neighbors of LXR |
|  | Neighbors of HDL |
|  | Neighbors of p38 |
|  | Neighbors of inflammatory cytokine |
|  | Neighbors of IL23 |
|  | Neighbors of estrogen receptor |
|  | Neighbors of T-cell receptor |
|  | Neighbors of histone deacetylase |
|  | Neighbors of PDGF-BB |
|  | Neighbors of IL1R |
|  | Neighbors of HIF-1 |
|  | Neighbors of GPCR |
|  | Neighbors of Notch |
|  | Neighbors of prostaglandin-endoperoxide synthase |
|  | Neighbors of heat shock protein 90 |
|  | Neighbors of lectin |
|  | Neighbors of aldehyde reductase |
|  | Neighbors of mitogen-activated protein kinase kinase |
|  | Neighbors of TSH |
|  | Neighbors of Ang II receptor |
|  | Neighbors of endothelin |
|  | Neighbors of NOS |
|  | Neighbors of superoxide dismutase |
|  | Neighbors of proteasome endopeptidase complex |
|  | Neighbors of class nuclear receptor with C4 zinc fingers |
|  | Neighbors of non-coding RNA |
|  | Neighbors of histone H3 |
|  | Neighbors of STAT5 |
|  | Neighbors of alpha2 adrenoceptor |
|  | Neighbors of vitronectin receptor (integrin) |
|  | Neighbors of PP2A |
|  | Neighbors of retinoid-X receptor subfamily |
|  | Neighbors of SAPK |
|  | Neighbors of WNT |
|  | Neighbors of gonadotropins |
|  | Neighbors of PP2B |
|  | Neighbors of phospholipase |
|  | Neighbors of AHR subfamily |
|  | Neighbors of ITG |
|  | Neighbors of endostatins |
|  | Neighbors of CD3 complex |
|  | Neighbors of TLR1/2 heterodimer |
|  | Neighbors of FSH |
|  | Neighbors of TORC1 |
|  | Neighbors of RA receptor |
|  | Neighbors of histone |
|  | Neighbors of Rho kinase |
|  | Neighbors of PDGF receptor |
|  | Neighbors of neuropeptides |
|  | Neighbors of histone acetyltransferase |
|  | Neighbors of adrenoceptor |
|  | Neighbors of phosphodiesterase |
|  | Neighbors of caspase |
|  | Neighbors of phosphoinositide phospholipase C |
|  | Neighbors of deacetylase |
|  | Neighbors of TLR4/Myd88 |
|  | 1\_Differentiation of psoriatic T cells |
|  | 1\_TNF and IL1B provoke ECM degradation in osteoarthritis |
|  | 1\_Local estrogen production stimulates ectopic endometrium proliferation |
|  | 3\_Enhanced angiogenesis in endometriosis |
|  | 4\_Endothelial cells repress vasoconstriction in hypertension |
|  | 3\_1\_Mucus accumulation in asthma |
|  | 4\_Mucin hyperproduction in goblet and mucous cells in COPD |
|  | 4\_1\_Polymorphisms associated with inflammatory bowel diseases |
|  | 1\_1\_Glutamate overload enforced prostaglandins and NO synthesis in neurons |
|  | drug-target pairs direct regulation |
|  | New Combined Pathway |
|  | Differentiation of psoriatic T cells |

---

|  |  |
| --- | --- |
| Group | Genes Hypermethylated in Melanoma |
|  | Genes with Mutations Associated with Vitiligo |
|  | Genes with Polymorphisms Associated with Migraine |

---

|  |  |
| --- | --- |
| MedScan ID | 5743 |

---

|  |  |
| --- | --- |
| LocusLink ID | 5743 |
|  | 19225 |
|  | 29527 |

---

|  |  |
| --- | --- |
| Alias | prostaglandin-endoperoxide synthase 2 (prostaglandin G/H synthase and cyclooxygenase) |
|  | Prostaglandin H2 synthase 2 |
|  | PHS II |
|  | prostaglandin G/H synthetase |
|  | 1DDX\_B |
|  | prostaglandin G/H synthetase and cyclooxygenase II |
|  | prostaglandin G/H synthase and cyclooxygenase II |
|  | GRIPGHS |
|  | prostaglandin-endoperoxide synthase 2 |
|  | PGHS2 |
|  | hCox II |
|  | Prostaglandin H2 synthetase 2 |
|  | PHS2 |
|  | PGHS II |
|  | OTTHUMP00000033524 |
|  | cyclooxygenase 2b |
|  | TIS10/PGS-2 |
|  | prostaglandin G/H synthase |
|  | ciclooxygenase II |
|  | Prostaglandin G/H synthase 2 |
|  | PGH synthase 2 |
|  | cyclooxygenase II |
|  | prostaglandin-endoperoxide synthetase 2 (prostaglandin G/H synthetase and cyclooxygenase) |
|  | prostaglandin G/H synthase and cyclooxygenase 2 |
|  | PES-2 |
|  | Prostaglandin H2 synthase II |
|  | glucocorticoid-regulated inflammatory cyclooxygenase |
|  | Prostaglandin H2 synthetase II |
|  | cyclooxygenase (COX)-2 |
|  | cyclo-oxygenase-2 |
|  | macrophage activation-associated marker protein P71/73 |
|  | PTGS2 |
|  | prostaglandin-endoperoxide synthetase 2 |
|  | Prostaglandin G/H synthetase 2 |
|  | prostaglandin-endoperoxide synthetase II |
|  | PGH synthetase 2 |
|  | cyclooxygenase 2 (COX-2) |
|  | PGS-2/TIS10 |
|  | hCox-2 |
|  | PGH synthetase II |
|  | prostaglandin G/H synthetase and cyclooxygenase 2 |
|  | TIS10 |
|  | ciclooxygenase-2 |
|  | cyclooxygenase 2 |
|  | prostaglandin-endoperoxide synthase II |
|  | 1DDX\_A |
|  | PGH synthase II |
|  | 1DDX\_Bs |
|  | 1DDX\_As |
|  | COX II |
|  | COX2 |
|  | COX-2 |
|  | PHS-2 |
|  | PGG/HS |
|  | PGHS-2 |
|  | cyclooxygenase-2 |
|  | PGHS IIs |
|  | hCox IIs |
|  | prostaglandin G/H synthase and cyclooxygenase |

---

|  |  |
| --- | --- |
| EC Number | 1.14.99.1 |

---

|  |  |
| --- | --- |
| GO ID | 0019899 |
|  | 0020037 |
|  | 0046872 |
|  | 0016702 |
|  | 0004601 |
|  | 0004666 |
|  | 0042803 |
|  | 0016740 |
|  | 0034356 |
|  | 0007568 |
|  | 0001525 |
|  | 0030282 |
|  | 0050873 |
|  | 0098869 |
|  | 0071318 |
|  | 0034644 |
|  | 0071498 |
|  | 0034605 |
|  | 0071456 |
|  | 0071284 |
|  | 0071260 |
|  | 0071471 |
|  | 0019371 |
|  | 0019221 |
|  | 0046697 |
|  | 0007566 |
|  | 0042633 |
|  | 0006954 |
|  | 0007612 |
|  | 0019372 |
|  | 0042759 |
|  | 0035633 |
|  | 0007613 |
|  | 0097756 |
|  | 0051926 |
|  | 0045786 |
|  | 0008285 |
|  | 0043154 |
|  | 1902219 |
|  | 0045986 |
|  | 0032227 |
|  | 0030728 |
|  | 0055114 |
|  | 0043065 |
|  | 0090336 |
|  | 0090050 |
|  | 0031622 |
|  | 0090271 |
|  | 0045429 |
|  | 0033138 |
|  | 0090362 |
|  | 0031394 |
|  | 0042307 |
|  | 0048661 |
|  | 0045987 |
|  | 0031915 |
|  | 0051968 |
|  | 0071636 |
|  | 0010575 |
|  | 0045907 |
|  | 0001516 |
|  | 0008217 |
|  | 0050727 |
|  | 0150077 |
|  | 1990776 |
|  | 0032355 |
|  | 0070542 |
|  | 0009750 |
|  | 0051384 |
|  | 0032496 |
|  | 0010226 |
|  | 0010042 |
|  | 0006979 |
|  | 0034612 |
|  | 0033280 |
|  | 0019233 |
|  | 0005901 |
|  | 0005737 |
|  | 0005783 |
|  | 0005788 |
|  | 0005789 |
|  | 0043005 |
|  | 0032991 |
|  | 0051213 |
|  | 0016491 |
|  | 0006633 |
|  | 0006631 |
|  | 0030216 |
|  | 0006629 |
|  | 0043066 |
|  | 0010942 |
|  | 0008284 |
|  | 0006693 |
|  | 0042127 |
|  | 0034097 |
|  | 0043231 |
|  | 0016020 |
|  | 0005634 |
|  | 0042493 |
|  | 0014070 |
|  | 0010033 |
|  | 0010243 |
|  | 0009314 |
|  | 0050473 |
|  | 0031090 |
|  | 0008289 |
|  | 0006928 |
|  | 0006769 |
|  | 0042346 |
|  | 0043234 |
|  | 0042640 |
|  | 0019674 |
|  | 0019369 |
|  | 0044281 |
|  | 0006766 |
|  | 0006767 |
|  | 0051726 |
|  | 0006805 |
|  | 0005635 |
|  | 0042446 |
|  | 0006692 |
|  | 0005792 |
|  | 0008610 |

---

|  |  |
| --- | --- |
| KEGG ID | hsa:5743 |
|  | mmu:19225 |
|  | rno:29527 |

---

|  |  |
| --- | --- |
| Organism | Homo sapiens {Organism urn:agi-taxid:9606} |
|  | Mus musculus {Organism urn:agi-taxid:10090} |
|  | Rattus norvegicus {Organism urn:agi-taxid:10116} |
|  | Homo sapiens |
|  | Mus musculus |
|  | Rattus norvegicus |

---

|  |  |
| --- | --- |
| Mouse chromosome position | 1 63.84 cM |
|  | 1 76.2 cM |

---

|  |  |
| --- | --- |
| OMIM ID | 600262 |

---

|  |  |
| --- | --- |
| Rat chromosome position | 13q21 |

---

|  |  |
| --- | --- |
| Hugo ID | 9605 |
|  | HGNC:9605 |

---

|  |  |
| --- | --- |
| Human chromosome position | 1q31.1 |
|  | 1q25.2-q25.3 |

---

|  |  |
| --- | --- |
| Swiss-Prot Accession | P35354 |
|  | P35354.2 |
|  | Q05769 |
|  | Q05769.1 |
|  | P35355 |
|  | P35355.1 |
|  | A8K802 |
|  | Q16876 |
|  | Q543K3 |
|  | Q64379 |
|  | Q925V4 |
|  | Q6ZYK7 |
|  | Q8IZA9 |
|  | Q9NNY7 |
|  | Q3UMR6 |
|  | Q7TMV2 |
|  | Q63124 |
|  | Q8CIP1 |

---

|  |  |
| --- | --- |
| PIR ID | A46150 |
|  | A49010 |
|  | JC2030 |

---

|  |  |
| --- | --- |
| GenBank ID | NC\_000001 |
|  | NM\_000963 |
|  | NP\_000954 |
|  | NG\_028206 |
|  | AF044206 |
|  | AF276953 |
|  | AAF85977 |
|  | AL033533 |
|  | AY229989 |
|  | AAO38056 |
|  | AY382629 |
|  | AAQ75702 |
|  | CH471067 |
|  | EAW91216 |
|  | D28235 |
|  | BAA05698 |
|  | HC025790 |
|  | CBH19826 |
|  | HI519548 |
|  | CBX54355 |
|  | KF455116 |
|  | L34209 |
|  | AAN87129 |
|  | U04636 |
|  | AAA57317 |
|  | U20548 |
|  | U44805 |
|  | AAD14842 |
|  | AJ634912 |
|  | CAG25548 |
|  | AK292167 |
|  | BAF84856 |
|  | AY151286 |
|  | AAN52932 |
|  | AY462100 |
|  | AAR23927 |
|  | BC013734 |
|  | AAH13734 |
|  | BM978796 |
|  | DA277667 |
|  | HM473188 |
|  | ADJ56342 |
|  | L15326 |
|  | AAA35803 |
|  | M90100 |
|  | AAA58433 |
|  | P35354 |
|  | NC\_000067 |
|  | NM\_011198 |
|  | NP\_035328 |
|  | AC114655 |
|  | AH002100 |
|  | AAA40448 |
|  | CH466520 |
|  | EDL39487 |
|  | AK049923 |
|  | BAC33986 |
|  | AK144726 |
|  | BAE26032 |
|  | AK144956 |
|  | BAE26154 |
|  | AK149820 |
|  | AK151293 |
|  | AK166221 |
|  | BAE38639 |
|  | AK172161 |
|  | BAE42855 |
|  | BC052900 |
|  | AAH52900 |
|  | BY033144 |
|  | CD547477 |
|  | M64291 |
|  | AAA39924 |
|  | M88242 |
|  | AAA37740 |
|  | M94967 |
|  | AAA39918 |
|  | X98792 |
|  | Q05769 |
|  | NC\_005112 |
|  | NM\_017232 |
|  | NP\_058928 |
|  | AC\_000081 |
|  | AABR07021355 |
|  | AAHX01075875 |
|  | CH473958 |
|  | EDM09586 |
|  | EDM09587 |
|  | AF159101 |
|  | AF233596 |
|  | AAF36986 |
|  | AY157736 |
|  | AAN52933 |
|  | FQ222702 |
|  | L20085 |
|  | AAA40947 |
|  | L25925 |
|  | AAA16477 |
|  | S67722 |
|  | AAB29401 |
|  | U03389 |
|  | AAA03466 |
|  | U04300 |
|  | AAA20246 |
|  | P35355 |
|  | NC\_018912 |
|  | AMYH02002314 |
|  | EU176282 |
|  | ABW03733 |
|  | EU176597 |
|  | ABW03398 |
|  | AC\_000023 |
|  | AAHY01012136 |
|  | M82862 |
|  | M82863 |
|  | M82864 |
|  | M82865 |
|  | M82866 |
|  | AC\_000133 |
|  | ABBA01027702 |
|  | XM\_006249971 |
|  | XP\_006250033 |
|  | AABR06075371 |
|  | CAB41240 |
|  | NT\_004487 |
|  | NW\_001838533 |
|  | NW\_926128 |
|  | AC\_000044 |
|  | Q6ZYK7 |
|  | Q8IZA9 |
|  | Q9NNY7 |
|  | NT\_078297 |
|  | NW\_001030662 |
|  | Q3UMR6 |
|  | Q543K3 |
|  | Q7TMV2 |
|  | NW\_001084685 |
|  | NW\_047397 |
|  | Q63124 |
|  | Q8CIP1 |

---

|  |  |
| --- | --- |
| Swiss-Prot ID | PGH2\_HUMAN |
|  | PGH2\_MOUSE |
|  | PGH2\_RAT |
|  | Q543K3\_MOUSE |
|  | A8K802\_HUMAN |

---

|  |  |
| --- | --- |
| Cell Localization | Microsome membrane |
|  | Endoplasmic reticulum membrane |
|  | Plasma membrane |

---

|  |  |
| --- | --- |
| Ensembl ID | ENSG00000073756 |
|  | ENSP00000356438.5 |
|  | ENST00000367468.10 |
|  | ENSMUSG00000032487 |
|  | ENSMUSP00000035065.7 |
|  | ENSMUST00000035065.8 |
|  | ENSRNOG00000002525 |
|  | ENSRNOP00000003567.2 |
|  | ENSRNOT00000003567.4 |
|  | ENST00000367468.9 |
|  | ENSP00000356438 |
|  | ENST00000367468 |
|  | ENSMUSP00000035065 |
|  | ENSMUST00000035065 |
|  | ENSRNOP00000003567 |
|  | ENSRNOT00000003567 |

---

|  |  |
| --- | --- |
| MGI ID | MGI:97798 |
|  | 97798 |

---

|  |  |
| --- | --- |
| RGD ID | 620349 |

---

|  |  |
| --- | --- |
| Unigene ID | Rn.44369 |
|  | Rn.217585 |
|  | Mm.292547 |
|  | Hs.196384 |

---

|  |  |
| --- | --- |
| Homologene ID | 31000 |

---

|  |  |
| --- | --- |
| IPI ID | IPI00197645 |
|  | IPI00308785 |
|  | IPI00018109 |
|  | IPI00447478 |
|  | IPI00816025 |
|  | IPI00462137 |

---
